# On the Protoxyde of Azote as an Anæsthetic Agent

**Published:** 1866-09

**Authors:** M. Préterre


BUFFALO

### Medical and Surgical Journal.

VOL. VI. SEPTEMBER, 1866. No. 2.

### Original Communications.

ART. I.—*On the Protoxyde of Azote as an Anæsthetic agent.* By M. Préterre. *Translated from the French.* BY C. C. F. GAY, M. D.

M. Ricord presented to the Society June 30th, 1866, in the name of M. Préterre, dentist, a *brochure* upon the employment of the protoxyde of azote as an anæsthetic agent, with the instrument designed for the inhalation of the gas. Desirous of being assured for ourselves of the value of a discovery that was thought to be destined to great future usefulness, says M. Prèterre, we have constructed an apparatus, described below, and we have undertaken a great number of experiments, the success of which have justified our expectations. We have constantly obtained, with the greatest rapidity, complete anæsthesia and of short duration. Quite recently we have extracted six teeth or roots, for a young lady extremely nervous, the operation was performed without pain, so that on waking the patient requested us to commence quickly. As all the observations we could report are similar, we confine ourselves within the limits of the description of that which passes when one submits an individual to the inhalation of the protoxyde of azote, with the aim of practicing an operation.

Anæsthesia is produced after one or two minutes of inspiration. Its duration, in general, is 30 to 50 seconds, which is sufficient time in order to practice a small operation, (ingrowing nail, extracting tooth, opening an abscess, etc.) In prolonging the inspiration of the gas we have obtained at one time three minutes of anæsthesia, but we have not tried to go beyond this. That which characterizes anæsthesia determined by the protoxyde of azote is the rapidity with which it is produced, and its short duration. We have quite recently put asleep a young man, extracted three molar teeth, and when he had awakened the time consumed was 130 seconds.

We wish not here to enter into the consideration of the mode of action of the protoxyde of azote. We only say it appears to us that the anæsthesia it produces is obtained so quickly that it has not the power to produce asphyxia as chloroform has. It appears to us probable that it produces upon the nervous system a special action, comparable to that of morphine and the other narcotics. We are convinced that the protoxyde of azote is an agent so useful as to be adopted very soon in France for minor surgical operations.

One hesitates often, and with reason, to submit a patient to the action of ether or chloroform for a minor operation, such as ingrowing toe-nails, opening abscess, etc., for one knows that anæsthesia, produced by these substances, has often been followed by death. The protoxyde of azote, on the contrary, presents, when it is employed perfectly pure, no danger. From the time it commenced to be studied, until now, that is to say, a period of six years, thousands of individuals have respired it without inconvenience. We have respired it several times withont being at all incommoded; it has been the same with all those persons to whom we have administered it.

In a single case we have seen, after an operation, an individual, prone to passing hallucination, wishing to escape from our hands, but that effect, produced so often, when one takes certain narcotic substances, such as stramonium or apocynum, is promptly dissipated. Protoxyde of azote, therefore, appears to us an anæsthetic agent extremely precious. Without doubt it cannot be substituted completely for ether and chloroform, but every day the practitioner will give preference to it in operations of short duration.

Here follows the description of the apparatus for administering the gas, which the translator omits.

The author passing successively in review the different processes of anæsthesia in use until now, in order to abolish pain, showed that all presented inconveniences more or less serious. Those employed to produce general anœsthesia were dangerous, and those in use for obtaining local anæsthesia inefficacious, while the protoxyde of azote does not produce the same inconveniences, and he lays down as a résumé of his labors the following conclusions:

1st.—Protoxyde of azote adds to the property of producing sleep very rapidly, anæsthesia of short duration.

2d.—When the gas is employed perfectly pure, it may be respired without danger, and never producing accident.

3d.—For all operations of short duration one must give to it preference to all known anæsthetic agents.

